# Morphology and Phylogeny Reveal Three New Species of *Cytospora* Associated with Tree Cankers in China

**DOI:** 10.3390/jof10020139

**Published:** 2024-02-09

**Authors:** Shuo Wang, Ning Jiang, Rong Ma

**Affiliations:** 1 College of Forestry and Horticulture, Xinjiang Agricultural University, Urumqi 830052, China; 15352572797@163.com; 2 Key Laboratory of Biodiversity Conservation of National Forestry and Grassland Administration, Ecology and Nature Conservation Institute, Chinese Academy of Forestry, Beijing 100091, China; n.jiang@caf.ac.cn

**Keywords:** Diaporthales, plant disease, systematics, taxonomy

## Abstract

*Cytospora* (Cytosporaceae, Diaporthales) is a fungal genus that usually inhabits plants as endophytes, saprobes, as well as pathogens. Species of this genus are characterized by possessing allantoid hyaline conidia and ascospores. Samples with typical Cytospora canker symptoms on *Prunus davidiana*, *P. padus* and *Salix* sp. were collected in Tibet and Xinjiang, China. Species were identified using both morphological and molecular approaches of combined loci of internal transcribed spacer region rDNA (ITS), the partial actin (*act*) region, RNA polymerase II second largest subunit (*rpb2*), the translation elongation factor 1-alpha (*tef1*) gene and the partial be-ta-tubulin (*tub2*) gene. Six isolates in the present study formed three distinct clades from previously known species. *Cytospora hejingensis* sp. nov. from *Salix* sp., *C. jilongensis* sp. nov. from *P. davidiana* and *C. kunsensis* from *P. padus* were proposed herein. The current study improves the understanding of species concept in *Cytospora*.

## 1. Introduction

*Cytospora* is a species-rich genus in family Cytosporaceae (order Diaporthales) and commonly inhabits plant tissues [1,2,3,4]. This genus was proposed in 1818 with four species, namely *C. betulina*, *C. epimyces*, *C. resinae* and *C. ribis* [5]. Another species *C. chrysosperma* was subsequently introduced [6] and later selected as the type species of this genus [7]. *Cytospora* can be different from the other diaporthalean genera by having allantoid hyaline conidia and ascospores [1,4,8,9,10].

Species of *Cytospora* were primarily identified and distinguished by their morphology and host [5,6,7]. However, recent studies employing molecular phylogeny revealed many cryptic species with similar morphology on the same host of known species of this genus [11,12,13,14,15]. For example, up to 28 *Cytospora* species were discovered from *Eucalyptus* spp. in South Africa with the help of DNA sequence evidence [2], eight from willow (*Salix* spp.) trees in China [16], six from *Castanea mollissima* in China [17], six from *Populus* hosts in China [18] and six from apple trees in Iran [19]. The taxonomy of *Cytospora* is currently more dependent on combined evidence of DNA sequence data, morphological features and ecology than species morphology and host associations [1,20].

Several species of *Cytospora* are reported to cause plant diseases including canker, wilt and dieback [21,22,23,24]. For example, *C. carpobroti* causes *Carpobrotus edulis* wilt disease in South Africa [21]; *C. oleicola* and *C. olivarum* are pathogenic to olives in the USA [22]; *C. parasitica* results in apple cankers in China [23]; and *Cytospora pistaciae* causes dieback and canker disease of pistachios in Italy [24]. There are still many cryptic species of *Cytospora* pathogenic to plants waiting for description.

In the present study, Cytospora canker symptoms were found from different tree hosts named *Prunus davidiana*, *P. padus* and *Salix* sp. in Tibet and Xinjiang, China. The aims of the present study were to identify the casual agents of the lesions, to introduce and describe new *Cytospora* species using both molecular and morphological approaches, and to discuss the species differences based on newly collected specimens.

## 2. Materials and Methods

### 2.1. Specimens and Strains

Investigations to collect fungal specimens were conducted in Tibet and Xinjiang during 2021 and 2022. During the surveys, dead and dying twigs and branches of tree hosts were checked manually, and then twigs and branches with obvious fungal fruiting bodies were recorded and collected. Samples were packed in paper bags and posted back for isolation.

Ascomata on branches of *Prunus padus* and *Salix* sp., and conidiomata on branches of *P. davidiana* were sectioned using sterile blades, and mucoid spore masses were removed and placed onto the surface of potato dextrose agar (PDA; potato, 200 g; glucose, 20 g; agar, 20 g; distilled water, to complete 1000 mL) media using sterile insect needles. Then, plates were incubated at 25 °C in darkness until spores germinated. Pieces of mycelium were cut and removed and placed onto a new PDA plate under a stereomicroscope to obtain the pure strains. Specimens and isolates were preserved in the China Forestry Culture Collection Center (CFCC; http://cfcc.caf.ac.cn/ (accessed on 2 January 2024)).

### 2.2. Morphological Observations

The *Cytospora* species observations were based on ascomata and conidiomata naturally formed on twigs and branches of *Prunus davidiana*, *P. padus* and *Salix* sp. The sexual and asexual fruiting bodies were sectioned using sterile blades and photographed using the Leica stereomicroscope (M205) (Leica Microsystems, Wetzlar, Germany). The asci, ascospores, conidiophores, conidiogenous cells and conidia were measured and photographed by a Nikon Eclipse 80i microscope (Nikon Corporation, Tokyo, Japan). The colony characteristics were observed and recorded on PDA plates at 25 °C in darkness.

### 2.3. DNA Extraction and Amplification

The total genomic DNA of *Cytospora* species were obtained from colonies growing on PDA plates by using the CTAB method [25]. The internal transcribed spacer region rDNA (ITS), the partial actin (*act*) region, RNA polymerase II second largest subunit (*rpb2*), the translation elongation factor 1-alpha (*tef1*) gene and the partial be-ta-tubulin (*tub2*) gene were amplified using primer pairs ITS1/ITS4, ACT512F/ACT783R, fRPB2-5f/fRPB2-7cR, 983F/2218R, Bt2a/Bt2b, respectively [26,27,28,29,30]. These regions were amplified as follows: an initial denaturation step of 5 min at 94 °C, followed by 35 cycles of 30 s at 94 °C, 50 s at 52 °C (ITS), 54°C (*tef1* and *tub2*), 55 °C (*rpb2*) or 58 °C (*act*), and 1 min at 72 °C, and a final elongation step of 7 min at 72 °C. The polymerase chain reaction products were sequenced using an ABI PRISM 3730XL DNA Analyser with a BigDye Terminator Kit v.3.1 (Invitrogen, Waltham, MA, USA) at the Shanghai Invitrogen Biological Technology Company Limited (Beijing, China).

### 2.4. Molecular Phylogeny

Sequences obtained in the present study were preliminarily identified by the BLAST search to confirm their classification. The referenced sequences of *Cytospora* were collected from recent publications (Table 1) and downloaded [1,24,25]. Strain CBS 160.32 (species *Diaporthe vaccinii*) was selected as the outgroup taxon. The five individual loci ITS, *act*, *rpb2*, *tef1* and *tub2* were aligned using MAFFT v. 6.0 and manually adjusted using MEGA v. 6.0 [31,32]. Then, five loci were combined and analyzed based on maximum likelihood (ML) and Bayes methods in the CIPRES Science Gateway platform [33]. The GTR substitution model was employed and 1000 non-parametric bootstrap replicates were set for ML phylogenic analysis. Four simultaneous Markov Chain runs for 1,000,000 generations were set during Bayesian analysis. The resulting trees were visualized in FigTree v. 1.4.0 and edited using Adobe Illustrator 2020.

## 3. Results

### 3.1. Phylogeny

**Figure 1:**
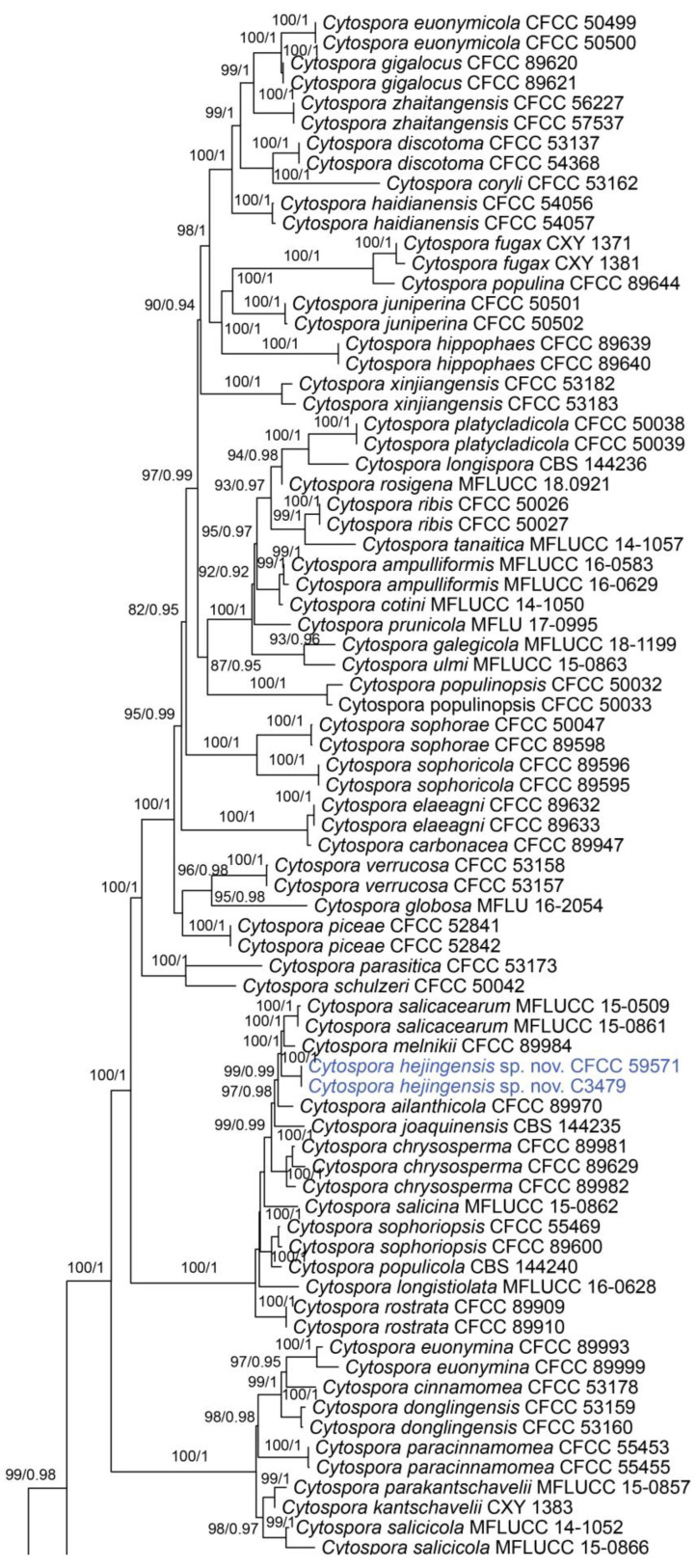

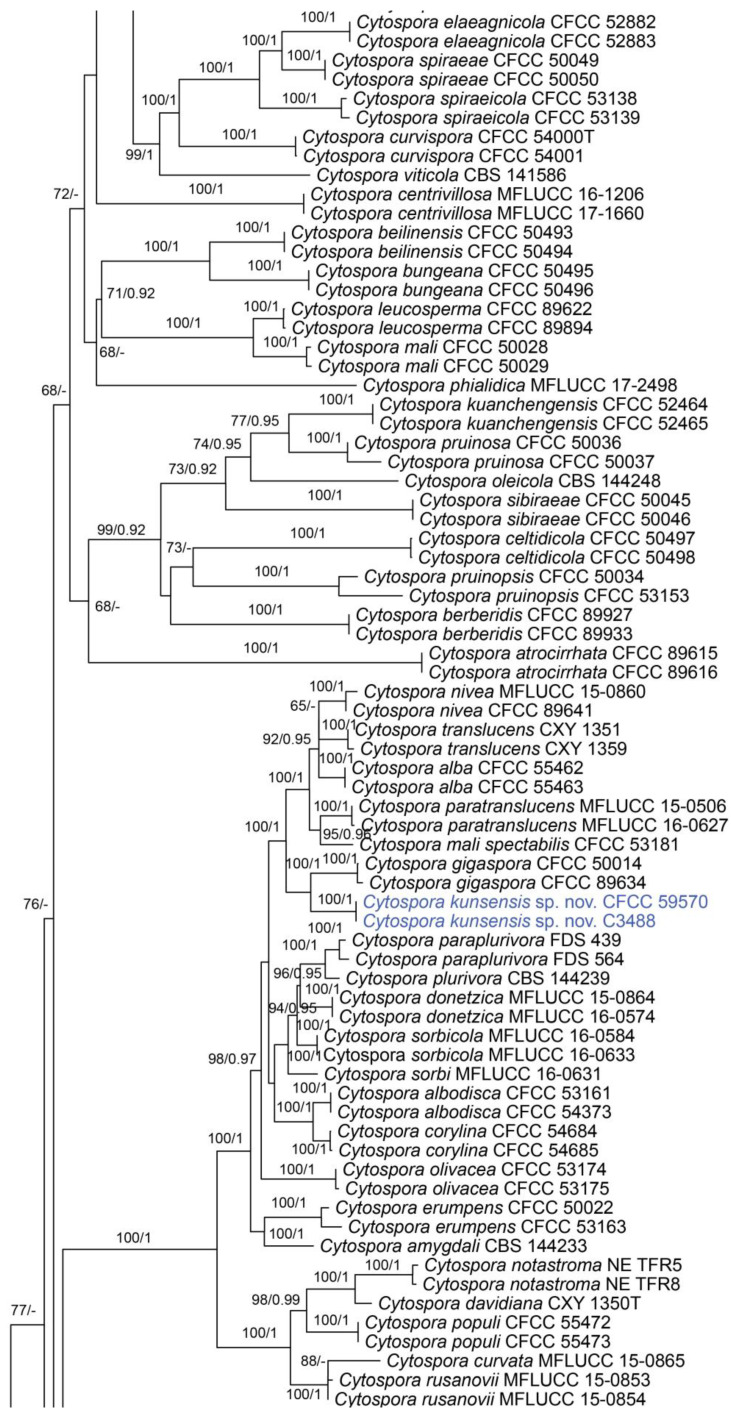

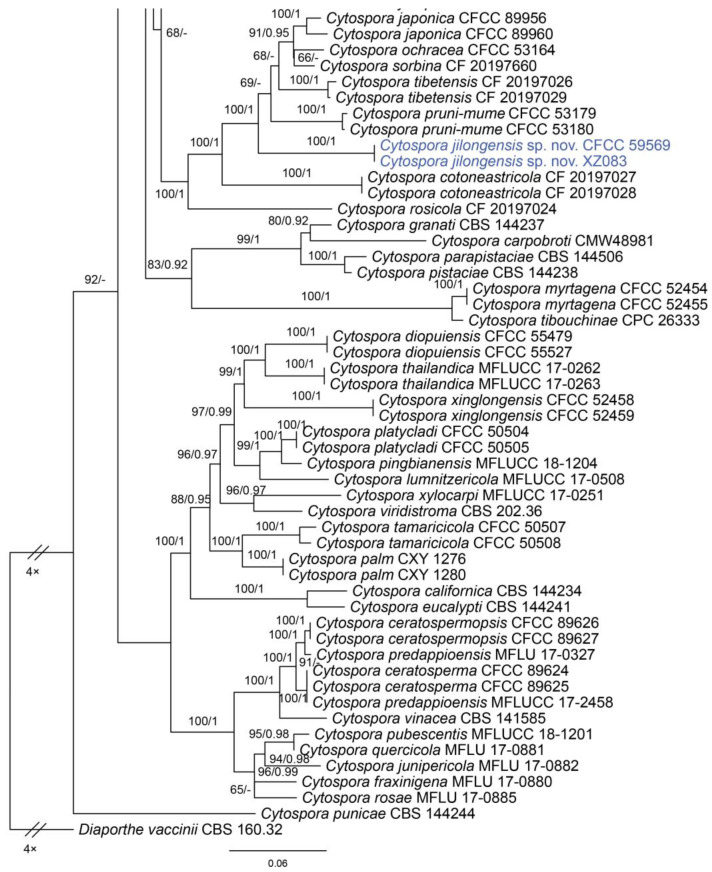
Phylogram of *Cytospora* resulting from a maximum likelihood analysis, based on a combined matrix of ITS, *act*, *rpb2*, *tef1* and *tub2*. Numbers above the branches indicate ML bootstraps (**left**, ML BS ≥ 50%) and Bayesian posterior probabilities (**right**, BPP ≥ 0.90). The tree is rooted with *Diaporthe vaccinii* (CBS 160.32). Isolates obtained from the present study are marked in blue.

In the phylogenetic analysis, the combined dataset of ITS, *act*, *rpb2*, *tef1* and *tub2* consisted of 202 strains. The final alignment comprised 2561 characters including 588 characters in ITS, 211 characters in *act*, 617 characters in *rpb2*, 536 characters in *tef1* and 609 characters *tub2*. The final ML optimization likelihood value of the best RAxML tree was −48,006.19, and the matrix had 1534 distinct alignment patterns, with 29.35% undetermined characters or gaps. Estimated base frequencies were as follows: A = 0.242905, C = 0.286434, G = 0.242250 and T = 0.228411; substitution rates AC = 1.374316, AG = 3.693698, AT = 1.471034, CG = 0.981185, CT = 6.248472 and GT = 1.0; and gamma distribution shape parameter α = 0.321644. The topology of our phylogenetic tree is nearly identical to previous publications. The topology of isolates from the present study in the RAxML and Bayesian analyses were congruent. Isolates CFCC 59571 and C3479 formed a distinct clade to CFCC 89984 (*C. melnikii*), MFLUCC 15-0509 and MFLUCC 15-0861 (*C. salicacearum*) with high support values (BS = 100, BPP = 1). Isolates CFCC 59570 and C3488 formed a clade close to CFCC 50014 and CFCC 89634 (*C. gigaspora*) with full support values (BS = 100, BPP = 1). Isolates CFCC 59570 and C3488 clustered together with CFCC 89956 and CFCC 89960 (*C. japonica*), CFCC 53164 (*C. ochracea*), CF 20197660 (*C. sorbina*), CF 20197026 and CF 20197029 (*C. tibetensis*), and CFCC 53179 and CFCC 53180 (*C. pruni-mume*) supported by high values (BS = 100, BPP = 1). Hence, six isolates from the present study formed three new clades distinct from previously known species named *Cytospora hejingensi* sp. nov., *C. jilongensis* sp. nov. and *C. kunsensis* sp. nov. (Figure 1).

### 3.2. Description of Cytospora hejingensis sp. nov. from Salix sp.

*Cytospora hejingensis* R. Ma & Ning Jiang, sp. nov. 

Figure 2.

MycoBank: MB851771

Etymology: named after the collection site of the holotype, Hejing County.

Description: Associated with branch and twig canker disease of *Salix* sp. Sexual morph: Ascostromata immersed in the bark, erumpent through the bark surface, scattered, (400–)650–900(–1250) μm diam., with 4–9 perithecia arranged irregularly. Conceptacle absent. Ectostromatic disc inconspicuous, usually surrounded by tightly aggregated ostiolar necks, (100–)150–250(–350) μm diam. Ostioles numerous, black, concentrated, arranged irregularly in a disc, (35–)50–65(–90) μm diam. Perithecia black, spherical, arranged circularly or irregularly, (120–)150–250(–300) μm diam. Asci free, clavate, (38–)45–70(–77) × (7–)8.5–10.5(–12.5) μm, 8-spored. Ascospores biseriate, allantoid, thin-walled, hyaline, aseptate, (6.5–)7–8(–9) × 2–2.5 μm. Asexual morph: undetermined. 

Culture characteristics: colonies on PDA flat, spreading, with flocculent mycelium, initially white to grey, secreting a dark green to black pigment in culture medium after 10 days, reaching a 90 mm diameter after 15 days at 25 °C in the dark.

Materials examined: China, Xinjiang Uygur Autonomous Region, Bayingolin Mongol Autonomous Prefecture, Hejing County, Kunse Forest Park, on cankered twigs and branches of *Salix* sp., 24 July 2021, Rong Ma (XJAU 3488, holotype); ex-type culture CFCC 59571; *ibid*. (culture C3488).

Notes: *Cytospora hejingensis* from *Salix* sp. in China is phylogenetically close to *C. melnikii* from *Malus domestica* in Russia and *C. salicacearum* from *Salix alba* in Russia (Figure 1). *C. hejingensis* is only known in sexual morph, and the other two species in asexual morph. Hence, it is impossible to compare them in morphology. However, *C. hejingensis* differs from *C. melnikii* and *C. salicacearum* by sequence data (22/560 in ITS, 35/211 in *act*, 36/617 in *rpb2* and 27/306 in *tef1* from *C. melnikii*; 25/560 in ITS, 37/211 in *act* and 26/617 in *rpb2* from *C. salicacearum*) [34].

**Figure 2:**
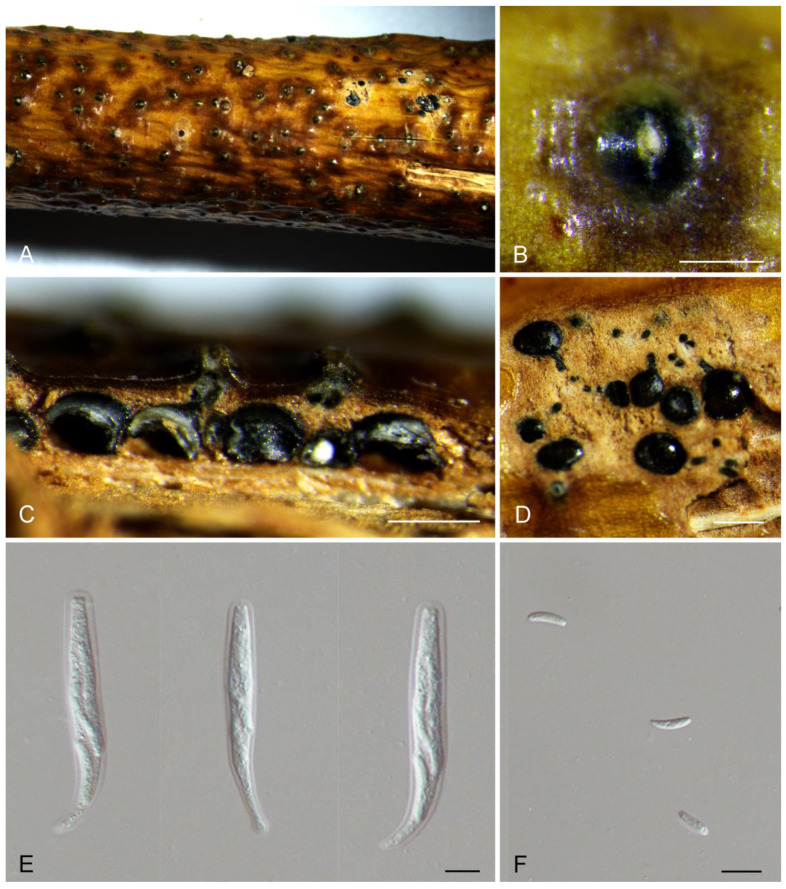
Morphology of *Cytospora hejingensis* from *Salix* sp. (**A**,**B**) Ascomata formed on branches. (**C**) Longitudinal section through the ascomata. (**D**) Transverse section of ascomata. (**E**) Asci. (**F**) Ascospores. Scale bars: (**B**) = 500 μm; (**C**) = 200 μm; (**D**) = 300 μm; (**E**,**F**) = 10 μm.

### 3.3. Description of Cytospora jilongensis sp. nov. from Prunus davidiana

*Cytospora jilongensis* R. Ma & Ning Jiang, sp. nov. 

Figure 3.

MycoBank: MB851772

Etymology: named after the collection site of the holotype, Jilong County.

Description: Associated with branch canker disease of *Prunus davidiana*. Sexual morph: undetermined. Asexual morph: Pycnidial stromata ostiolated, semi-immersed in the host bark, scattered, pulvinate, with multiple locules. Conceptacle dark brown, circular surrounded stromata. Ectostromatic grey, circular to ovoid, (100–)180–240(–370) μm diam., with one ostiole per disc. Ostioles dark, at the same level as the disc, (30–)50–75(–95) μm diam. Locule numerous, arranged circularly or elliptically with independent walls, (250–)400–500(–750) μm diam. Peridium comprising few layers of cells of textura angularis, brown to dark brown. Conidiophores hyaline, branched, thin-walled, filamentous. Conidiogenous cells enteroblastic polyphialidic, 7.5–18.5 × 1.5–2.5 μm. Conidia hyaline, allantoid, smooth, aseptate, thin-walled, (9.3–)10.2–11.6(–12.5) × 2.6–3.2 μm.

Culture characteristics: colonies on PDA flat, spreading, with moderate flocculent mycelium, initially white, becoming orange after 10 days, reaching a 90 mm diameter after 25 days at 25 °C in the dark.

Materials examined: China, Tibet Tibetan Autonomous Region, Shigatse City, Jilong County, Jilong Town, on cankered branches of *Prunus davidiana*, 12 August 2022, Jin Peng, Jiang Ning and Liu Min (CAF800087, holotype); ex-type culture CFCC 59569; *ibid*. (culture XZ083).

Notes: *Cytospora jilongensis* from *Prunus davidiana* is phylogenetically close to *C. japonica* from *P. cerasifera* and *P. persica*, *C. ochracea* from *Cotoneaster* sp., *C. sorbina* from *Sorbus tianschanica*, *C. tibetensis* from *Cotoneaster* sp. and *C. pruni-mume* from *Prunus mume* (Figure 1). However, *C. jilongensis* (10.2–11.6 × 2.6–3.2 μm) differs from *C. japonica* (6.5–8.5 × 1.5–2 μm), *C. ochracea* (8.5–9.0 × 1.5–2.5 μm), *C. sorbina* (4.5–5.5 × 1–1.5 μm), *C. tibetensis* (5.0–5.5 × 1.5–2 μm) and *C. pruni-mume* (5.5–6.5 × 1.5–2 μm) in conidial size and hosts [1,14].

**Figure 3:**
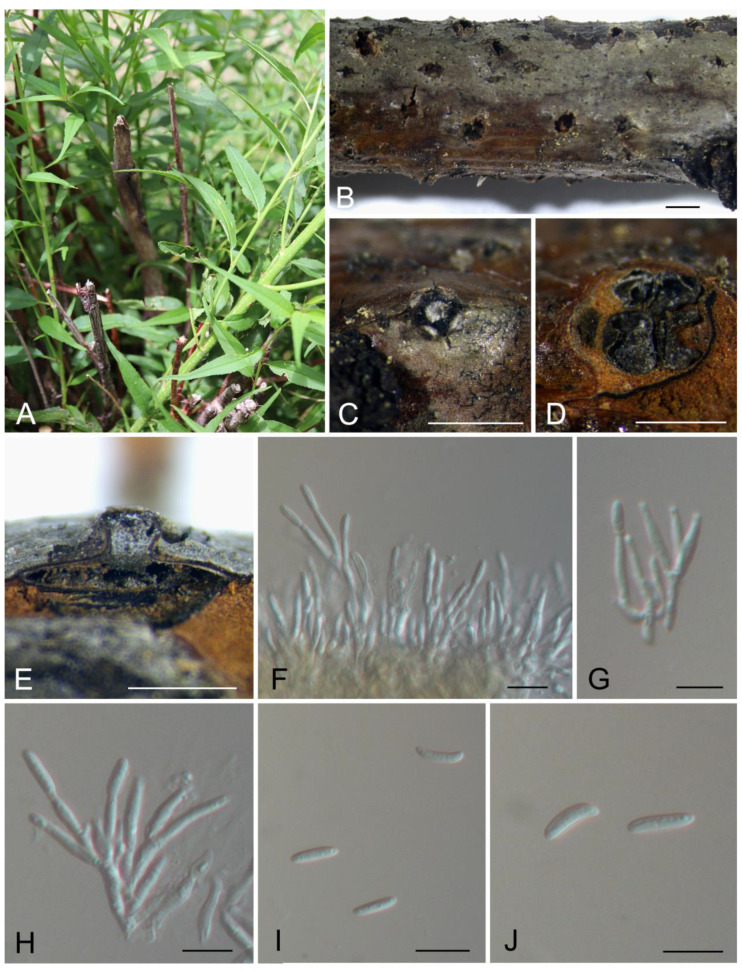
Morphology of *Cytospora jilongensis* from *Prunus davidiana*. (**A**) Symptoms of canker disease on the host. (**B**,**C**) Conidiomata formed on branches. (**D**) Transverse section through the conidioma. (**E**) Longitudinal section through the conidioma. (**F**–**H**) Conidiophores and conidiogenous cells. (**I**,**J**) Conidia. Scale bars: (**B**) = 2 mm; (**C**,**D**) = 1 mm; (**E**) = 800 μm; (**F**–**J**) = 10 μm.

### 3.4. Description of Cytospora kunsensis sp. nov. from Prunus padus

*Cytospora kunsensis* R. Ma & Ning Jiang, sp. nov. 

Figure 4.

MycoBank: MB851773

Etymology: named after the collection site of the holotype, Kunse Forest Park.

Description: Associated with branch and twig canker disease of *Prunus padus*. Sexual morph: Ascostromata immersed in the bark, erumpent through the bark surface, scattered, (750–)950–1100(–1350) μm diam., with 5–11 perithecia arranged circularly. Conceptacle absent. Ectostromatic disc white, surrounded by tightly aggregated ostiolar necks, (100–)150–300(–350) μm diam. Ostioles numerous, black, concentrated, arranged circularly in a disc, (40–)50–75(–90) μm diam. Perithecia black, spherical, arranged circularly or irregularly, (180–)250–350(–420) μm diam. Asci free, clavate, (38–)48–80(–86) × (7.5–)9–12(–13.5) μm, eight-spored. Ascospores biseriate, allantoid, thin-walled, hyaline, aseptate, (10–)12.5–17(–19.5) × 2–2.5 μm. Asexual morph: undetermined. 

Culture characteristics: colonies on PDA flat, spreading, with flocculent mycelium, white, with a dark grey color in the center, fast growing, reaching a 90 mm diameter after 7 days and forming abundant black ascomata after 25 days at 25 °C.

Materials examined: China, Xinjiang Uygur Autonomous Region, Bayingolin Mongol Autonomous Prefecture, Hejing County, Kunse Forest Park, on cankered twigs and branches of *Prunus padus*, 24 July 2021, Rong Ma (XJAU 3479, holotype); ex-type culture CFCC 59570; *ibid*. (culture C3479).

Notes: *Cytospora kunsensis* from *Prunus padus* is phylogenetically close to *C. gigaspora* from *Salix psammophila* (Figure 1). However, *C. kunsensis* can be distinguished from *C. gigaspora* by sequence data (19/548 in ITS, 32/211 in *act*, 56/617 in *rpb2*, 36/303 in *tef1* and 42/421 in *tub2*) [11].

**Figure 4:**
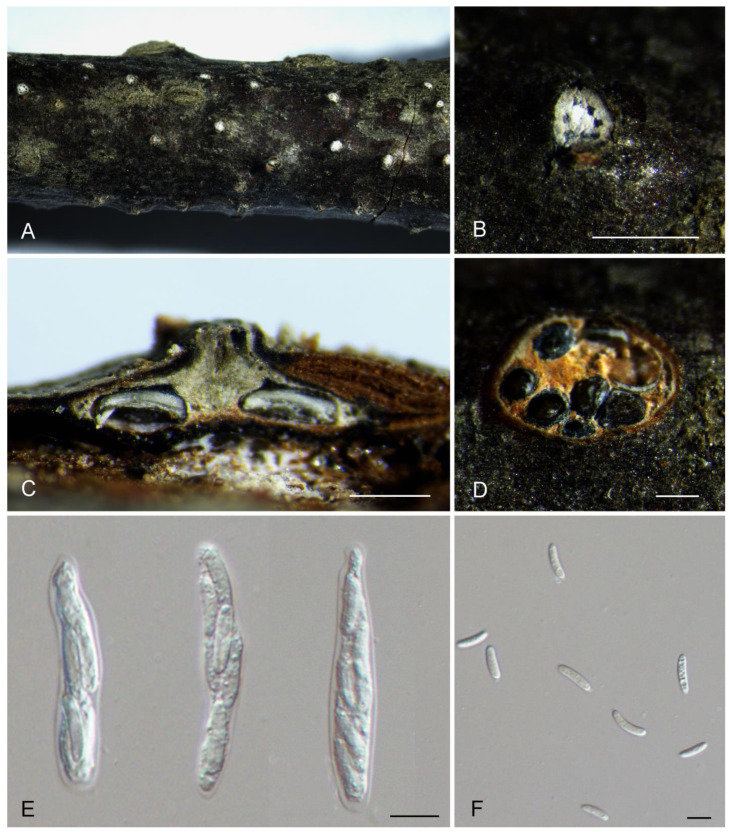
Morphology of *Cytospora kunsensis* from *Prunus padus*. (**A**,**B**) Ascomata formed on branches. (**C**) Longitudinal section through the ascomata. (**D**) Transverse section of ascomata. (**E**) Asci. (**F**) Ascospores. Scale bars: (**B**) = 500 μm; (**C**,**D**) = 300 μm; (**E**,**F**) = 10 μm.

## 4. Discussion

In the present study, samples of *Cytospora* with fruiting bodies were collected from Xinjiang and Tibet, and identified based on both morphological and phylogenetical approaches of combined ITS, *act*, *rpb2*, *tef1* and *tub2* loci. We proposed three new species, i.e., *Cytospora hejingensis* sp. nov. from *Salix* sp., *C. jilongensis* sp. nov. from *P. davidiana* and *C. kunsensis* from *P. padus*.

Of the new species introduced in the current study, two taxa (*C. jilongensis* and *C. kunsensis*) were isolated from the plant genus *Prunus*. Hence, a total of nine species of *Cytospora* were found in host genus *Prunus*, where the previous seven species are *C. cinnamomea*, *C. erumpens*, *C. japonica*, *C. leucostoma*, *C. olivacea*, *C. populinopsis* and *C. pruni-mume* [14]. *C. kunsensis* is distinguished from *C. populinopsis* in eight-spored asci, and these two species are only known in sexual morph [1]. The other seven species are known in asexual species with similar conidial morphology but different sequence data of ITS, *act*, *rpb2*, *tef1* and *tub2* loci. The example of *Cytospora* species from *Prunus* implies that DNA sequence data are necessary to separate species during pathogen identifications.

Another example is the *Cytospora* species from the host genus *Salix*. Until now, over 10 species of *Cytospora* were discovered from the host genus *Salix*, including one species *Cytospora hejingensis* introduced in the current study [35]. Most of them are confirmed to be pathogens associated with canker diseases [35]. The new species from the present study needs a pathogenicity test to evaluate its virulence to willow trees in the future.

In the traditional classification and identification of species in *Cytospora*, spore morphology and host information are the most important evidence to identify *Cytospora* species [5,6,7]. However, by using the molecular data, many cryptic species with the same hosts and similar spore morphology were recently revealed [1,14,15,35]. The molecular classification system for *Cytospora* based on morphology, phylogeny and host information is more scientific than that mainly based on morphology before. 

## Figures and Tables

**Table 1:** Strains and their GenBank accession numbers used in this study.

Species	Strain	GenBank Accession Numbers				
		ITS	*act*	*rpb2*	*tef1*	*tub2*
*Cytospora ailanthicola*	CFCC 89970	MH933618	MH933526	MH933592	MH933494	MH933565
*Cytospora albodisca*	CFCC 53161	MW418406	MW422899	MW422909	MW422921	MW422933
*Cytospora albodisca*	CFCC 54373	MW418407	MW422900	MW422910	MW422922	MW422934
*Cytospora alba*	CFCC 55462^T^	NR182387	OK303457	OK303516	OK303577	OK303644
*Cytospora alba*	CFCC 55463	MZ702596	OK303458	OK303517	OK303578	OK303645
*Cytospora ampulliformis*	MFLUCC 16-0583^T^	KY417726	KY417692	KY417794		
*Cytospora ampulliformis*	MFLUCC 16-0629	KY417727	KY417693	KY417795		
*Cytospora amygdali*	CBS 144233^T^	MG971853	MG972002		MG971659	
*Cytospora atrocirrhata*	CFCC 89615	KR045618	KF498673	KU710946	KP310858	KR045659
*Cytospora atrocirrhata*	CFCC 89616	KR045619	KF498674	KU710947	KP310859	KR045660
*Cytospora beilinensis*	CFCC 50493^T^	MH933619	MH933527		MH933495	MH933561
*Cytospora beilinensis*	CFCC 50494	MH933620	MH933528		MH933496	MH933562
*Cytospora berberidis*	CFCC 89927^T^	KR045620	KU710990	KU710948	KU710913	KR045661
*Cytospora berberidis*	CFCC 89933	KR045621	KU710991	KU710949	KU710914	KR045662
*Cytospora bungeana*	CFCC 50495^T^	MH933621	MH933529	MH933593	MH933497	MH933563
*Cytospora bungeana*	CFCC 50496	MH933622	MH933530	MH933594	MH933498	MH933564
*Cytospora californica*	CBS 144234^T^	MG971935	MG972083		MG971645	
*Cytospora carbonacea*	CFCC 89947	KR045622	KP310842	KU710950	KP310855	KP310825
*Cytospora carpobroti*	CMW48981^T^	MH382812			MH411212	MH411207
*Cytospora celtidicola*	CFCC 50497^T^	MH933623	MH933531	MH933595	MH933499	MH933566
*Cytospora celtidicola*	CFCC 50498	MH933624	MH933532	MH933596	MH933500	MH933567
*Cytospora centrivillosa*	MFLUCC 16-1206^T^	MF190122		MF377600		
*Cytospora centrivillosa*	MFLUCC 17-1660	MF190123		MF377601		
*Cytospora ceratosperma*	CFCC 89624	KR045645		KU710976	KP310860	KR045686
*Cytospora ceratosperma*	CFCC 89625	KR045646		KU710977	KP310861	KR045687
*Cytospora ceratospermopsis*	CFCC 89626^T^	KR045647	KU711011	KU710978	KU710934	KR045688
*Cytospora ceratospermopsis*	CFCC 89627	KR045648	KU711012	KU710979	KU710935	KR045689
*Cytospora chrysosperma*	CFCC 89629	KF765673		KF765705		
*Cytospora chrysosperma*	CFCC 89981	MH933625	MH933533	MH933597	MH933501	MH933568
*Cytospora chrysosperma*	CFCC 89982	KP281261	KP310835		KP310848	KP310818
*Cytospora cinnamomea*	CFCC 53178^T^	MK673054	MK673024			MK672970
*Cytospora coryli*	CFCC 53162^T^	MN854450		MN850751	MN850758	MN861120
*Cytospora corylina*	CFCC 54684^T^	MW839861	MW815937	MW815951	MW815886	MW883969
*Cytospora corylina*	CFCC 54685	MW839862	MW815938	MW815952	MW815887	MW883970
*Cytospora cotini*	MFLUCC 14-1050^T^	KX430142		KX430144		
*Cytospora cotoneastricola*	CF 20197027	MK673072	MK673042	MK673012	MK672958	MK672988
*Cytospora cotoneastricola*	CF 20197028	MK673073	MK673043	MK673013	MK672959	MK672989
*Cytospora curvata*	MFLUCC 15-0865^T^	KY417728	KY417694			
*Cytospora curvispora*	CFCC 54000^T^	MW839851	MW815931	MW815945	MW815880	MW883963
*Cytospora curvispora*	CFCC 54001	MW839853	MW815932	MW815946	MW815881	MW883964
*Cytospora davidiana*	CXY 1350^T^	KM034870				
*Cytospora diopuiensis*	CFCC 55479	OQ344753	OQ410625	OQ398735	OQ398762	OQ398791
*Cytospora diopuiensis*	CFCC 55527	OQ344754	OQ410626	OQ398736	OQ398763	OQ398792
*Cytospora discotoma*	CFCC 53137^T^	MW418404	MW422897	MW422907	MW422919	MW422931
*Cytospora discotoma*	CFCC 54368	MW418405	MW422898	MW422908	MW422920	MW422932
*Cytospora donetzica*	MFLUCC 15-0864	KY417729	KY417695	KY417797		
*Cytospora donetzica*	MFLUCC 16-0574^T^	KY417731	KY417697	KY417799		
*Cytospora donglingensis*	CFCC 53159^T^	MW418412	MW422903	MW422915	MW422927	MW422939
*Cytospora donglingensis*	CFCC 53160	MW418414	MW422905	MW422917	MW422929	MW422941
*Cytospora elaeagni*	CFCC 89632	KR045626	KU710995	KU710955	KU710918	KR045667
*Cytospora elaeagni*	CFCC 89633	KF765677	KU710996	KU710956	KU710919	KR045668
*Cytospora elaeagnicola*	CFCC 52882^T^	MK732341	MK732344	MK732347		
*Cytospora elaeagnicola*	CFCC 52883	MK732342	MK732345	MK732348		
*Cytospora erumpens*	CFCC 50022	MH933627	MH933534		MH933502	MH933569
*Cytospora erumpens*	CFCC 53163	MK673059	MK673029	MK673000	MK672948	MK672975
*Cytospora eucalypti*	CBS 144241	MG971907	MG972056		MG971617	
*Cytospora euonymicola*	CFCC 50499^T^	MH933628	MH933535	MH933598	MH933503	MH933570
*Cytospora euonymicola*	CFCC 50500	MH933629	MH933536	MH933599	MH933504	MH933571
*Cytospora euonymina*	CFCC 89993^T^	MH933630	MH933537	MH933600	MH933505	MH933590
*Cytospora euonymina*	CFCC 89999	MH933631	MH933538	MH933601	MH933506	MH933591
*Cytospora fraxinigena*	MFLU 17-0880^T^	NR154859				
*Cytospora fugax*	CXY 1371	KM034852				KM034891
*Cytospora fugax*	CXY 1381	KM034853				KM034890
*Cytospora fusispora*	NFCCI 4372	MN227694				
*Cytospora galegicola*	MFLUCC 18-1199^T^	MK912128	MN685810	MN685820		
*Cytospora gigalocus*	CFCC 89620^T^	KR045628	KU710997	KU710957	KU710920	KR045669
*Cytospora gigalocus*	CFCC 89621	KR045629	KU710998	KU710958	KU710921	KR045670
*Cytospora gigaspora*	CFCC 50014	KR045630	KU710999	KU710959	KU710922	KR045671
*Cytospora gigaspora*	CFCC 89634^T^	KF765671	KU711000	KU710960	KU710923	KR045672
*Cytospora globosa*	MFLU 16-2054^T^	MT177935		MT432212	MT454016	
*Cytospora granati*	CBS 144237^T^	MG971799	MG971949		MG971514	
*Cytospora haidianensis*	CFCC 54056	MT360041	MT363978	MT363987	MT363997	MT364007
*Cytospora haidianensis*	CFCC 54057^T^	MT360042	MT363979	MT363988	MT363998	MT364008
***Cytospora hejingensis***	**CFCC 59571^T^**	**PP060455**	**PP059657**	**PP059663**	**PP059667**	**PP059673**
***Cytospora hejingensis***	**C3479**	**PP060456**	**PP059658**	**PP059664**	**PP059668**	**PP059674**
*Cytospora hippophaës*	CFCC 89639	KR045632	KU711001	KU710961	KU710924	KR045673
*Cytospora hippophaës*	CFCC 89640	KF765682	KF765730	KU710962	KP310865	KR045674
*Cytospora japonica*	CFCC 89956	KR045624	KU710993	KU710953	KU710916	KR045665
*Cytospora japonica*	CFCC 89960	KR045625	KU710994	KU710954	KU710917	KR045666
***Cytospora jilongensis***	**CFCC 59569^T^**	**PP060457**	**PP059659**		**PP059669**	**PP059675**
***Cytospora jilongensis***	**XZ083**	**PP060458**	**PP059660**		**PP059670**	**PP059676**
*Cytospora joaquinensis*	CBS 144235	MG971895	MG972044		MG971605	
*Cytospora junipericola*	MFLU 17-0882^T^	MF190125			MF377580	
*Cytospora juniperina*	CFCC 50501^T^	MH933632	MH933539	MH933602	MH933507	
*Cytospora juniperina*	CFCC 50502	MH933633	MH933540	MH933603	MH933508	MH933572
*Cytospora kantschavelii*	CXY 1383	KM034867				
*Cytospora kuanchengensis*	CFCC 52464^T^	MK432616	MK442940	MK578076		
*Cytospora kuanchengensis*	CFCC 52465	MK432617	MK442941	MK578077		
***Cytospora kunsensis***	**CFCC 59570^T^**	**PP060459**	**PP059661**	**PP059665**	**PP059671**	**PP059677**
***Cytospora kunsensis***	**C3488**	**PP060460**	**PP059662**	**PP059666**	**PP059672**	**PP059678**
*Cytospora leucosperma*	CFCC 89622	KR045616	KU710988	KU710944	KU710911	KR045657
*Cytospora leucosperma*	CFCC 89894	KR045617	KU710989	KU710945	KU710912	KR045658
*Cytospora longispora*	CBS 144236^T^	MG971905	MG972054	NA	MG971615	NA
*Cytospora longistiolata*	MFLUCC 16-0628	KY417734	KY417700	KY417802	NA	NA
*Cytospora lumnitzericola*	MFLUCC 17-0508^T^	MG975778	MH253457	MH253461	NA	NA
*Cytospora mali*	CFCC 50028	MH933641	MH933548	MH933606	MH933513	MH933577
*Cytospora mali*	CFCC 50029	MH933642	MH933549	MH933607	MH933514	MH933578
*Cytospora mali-spectabilis*	CFCC 53181^T^	MK673066	MK673036	MK673006	MK672953	MK672982
*Cytospora melnikii*	CFCC 89984	MH933644	MH933551	MH933609	MH933515	MH933580
*Cytospora myrtagena*	CFCC 52454	MK432614	MK442938	MK578074		
*Cytospora myrtagena*	CFCC 52455	MK432615	MK442939	MK578075		
*Cytospora nivea*	MFLUCC 15-0860	KY417737	KY417703	KY417805		
*Cytospora nivea*	CFCC 89641	KF765683	KU711006	KU710967	KU710929	KR045679
*Cytospora notastroma*	NE_TFR5	JX438632			JX438543	
*Cytospora notastroma*	NE_TFR8	JX438633			JX438542	
*Cytospora ochracea*	CFCC 53164^T^	MK673060	MK673030	MK673001	MK672949	MK672976
*Cytospora oleicola*	CBS 144248^T^	MG971944	MG972098		MG971660	
*Cytospora olivacea*	CFCC 53174	MK673058	MK673028	MK672999		MK672974
*Cytospora olivacea*	CFCC 53175	MK673062	MK673032	MK673003		MK672978
*Cytospora palm*	CXY 1276	JN402990			KJ781296	
*Cytospora palm*	CXY 1280^T^	JN411939			KJ781297	
*Cytospora paracinnamomea*	CFCC 55453^T^	MZ702594	OK303456	OK303515	OK303576	OK303643
*Cytospora paracinnamomea*	CFCC 55455^T^	MZ702598	OK303460	OK303519	OK303580	OK303647
*Cytospora parakantschavelii*	MFLUCC 15-0857^T^	KY417738	KY417704	KY417806		
*Cytospora parapistaciae*	CBS 144506^T^	MG971804	MG971954		MG971519	
*Cytospora paraplurivora*	FDS-439	OL640182	OL631586		OL631589	
*Cytospora paraplurivora*	FDS-564^T^	OL640183	OL631587		OL631590	
*Cytospora parasitica*	CFCC 53173	MK673070	MK673040	MK673010	MK672957	MK672986
*Cytospora paratranslucens*	MFLUCC 15-0506^T^	KY417741	KY417707	KY417809		
*Cytospora paratranslucens*	MFLUCC 16-0627	KY417742	KY417708	KY417810		
*Cytospora phialidica*	MFLUCC 17-2498	MT177932		MT432209	MT454014	
*Cytospora piceae*	CFCC 52841^T^	MH820398	MH820406	MH820395	MH820402	MH820387
*Cytospora piceae*	CFCC 52842	MH820399	MH820407	MH820396	MH820403	MH820388
*Cytospora pingbianensis*	MFLUCC 18-1204^T^	MK912135	MN685817	MN685826		
*Cytospora pistaciae*	CBS 144238^T^	MG971802	MG971952		MG971517	
*Cytospora platycladi*	CFCC 50504^T^	MH933645	MH933552	MH933610	MH933516	MH933581
*Cytospora platycladi*	CFCC 50505	MH933646	MH933553	MH933611	MH933517	MH933582
*Cytospora platycladicola*	CFCC 50038^T^	KT222840	MH933555	MH933613	MH933519	MH933584
*Cytospora platycladicola*	CFCC 50039	KR045642	KU711008	KU710973	KU710931	KR045683
*Cytospora plurivora*	CBS 144239^T^	MG971861	MG972010		MG971572	
*Cytospora populi*	CFCC 55472^T^	MZ702609	OK303471	OK303530	OK303591	OK303658
*Cytospora populi*	CFCC 55473	MZ702610	OK303472	OK303531	OK303592	OK303659
*Cytospora populicola*	CBS 144240	MG971891	MG972040		MG971601	
*Cytospora populina*	CFCC 89644^T^	KF765686	KU711007	KU710969	KU710930	KR045681
*Cytospora populinopsis*	CFCC 50032^T^	MH933648	MH933556	MH933614	MH933520	MH933585
*Cytospora populinopsis*	CFCC 50033	MH933649	MH933557	MH933615	MH933521	MH933586
*Cytospora predappioensis*	MFLUCC 17-2458^T^	MG873484				
*Cytospora predappioensis*	MFLU 17-0327	MH253451	MH253449	MH253450		
*Cytospora prunicola*	MFLU 17-0995^T^	MG742350	MG742353	MG742352		
*Cytospora pruni-mume*	CFCC 53179	MK673057	MK673027		MK672947	MK672973
*Cytospora pruni-mume*	CFCC 53180^T^	MK673067	MK673037	MK673007	MK672954	MK672983
*Cytospora pruinopsis*	CFCC 50034^T^	KP281259	KP310836	KU710970	KP310849	KP310819
*Cytospora pruinopsis*	CFCC 53153	MN854451	MN850763	MN850752	MN850759	MN861121
*Cytospora pruinosa*	CFCC 50036	KP310800	KP310832		KP310845	KP310815
*Cytospora pruinosa*	CFCC 50037	MH933650	MH933558		MH933522	MH933589
*Cytospora pubescentis*	MFLUCC 18-1201^T^	MK912130	MN685812	MN685821		
*Cytospora punicae*	CBS 144244	MG971943	MG972091		MG971654	
*Cytospora quercicola*	MFLU 17-0881	MF190128				
*Cytospora ribis*	CFCC 50026	KP281267	KP310843	KU710972	KP310856	KP310826
*Cytospora ribis*	CFCC 50027	KP281268	KP310844		KP310857	KP310827
*Cytospora rosae*	MFLU 17-0885	MF190131				
*Cytospora rosicola*	CF 20197024^T^	MK673079	MK673049	MK673019	MK672965	MK672995
*Cytospora rosigena*	MFLUCC 18-0921^T^	MN879872				
*Cytospora rostrata*	CFCC 89909	KR045643	KU711009	KU710974	KU710932	KR045684
*Cytospora rostrata*	CFCC 89910	KR045644	KU711010	KU710975	KU710933	
*Cytospora rusanovii*	MFLUCC 15-0853	KY417743	KY417709	KY417811		
*Cytospora rusanovii*	MFLUCC 15-0854^T^	KY417744	KY417710	KY417812		
*Cytospora salicacearum*	MFLUCC 15-0509	KY417746	KY417712	KY417814		
*Cytospora salicacearum*	MFLUCC 15-0861	KY417745	KY417711	KY417813		
*Cytospora salicicola*	MFLUCC 14-1052^T^	KU982636	KU982637			
*Cytospora salicicola*	MFLUCC 15-0866	KY417749	KY417715	KY417817		
*Cytospora salicina*	MFLUCC 15-0862	KY417750	KY417716	KY417818		
*Cytospora salicina*	MFLUCC 16-0637	KY417751	KY417717	KY417819		
*Cytospora schulzeri*	CFCC 50042	KR045650	KU711014	KU710981	KU710937	KR045691
*Cytospora sibiraeae*	CFCC 50045^T^	KR045651	KU711015	KU710982	KU710938	KR045692
*Cytospora sibiraeae*	CFCC 50046	KR045652	KU711016	KU710983	KU710939	KR045693
*Cytospora sophorae*	CFCC 50047	KR045653	KU711017	KU710984	KU710940	KR045694
*Cytospora sophorae*	CFCC 89598	KR045654	KU711018	KU710985	KU710941	KR045695
*Cytospora sophoricola*	CFCC 89596	KR045656	KU711020	KU710987	KU710943	KR045697
*Cytospora sophoricola*	CFCC 89595^T^	KR045655	KU711019	KU710986	KU710942	KR045696
*Cytospora sophoriopsis*	CFCC 55469	MZ702583	OK303445	OK303504	OK303565	OK303632
*Cytospora sophoriopsis*	CFCC 89600	KR045623	KU710992	KU710951	KU710915	KP310817
*Cytospora sorbi*	MFLUCC 16-0631^T^	KY417752	KY417718	KY417820		
*Cytospora sorbicola*	MFLUCC 16-0584^T^	KY417755	KY417721	KY417823		
*Cytospora sorbicola*	MFLUCC 16-0633	KY417758	KY417724	KY417826		
*Cytospora sorbina*	CF 20197660^T^	MK673052	MK673022		MK672943	MK672968
*Cytospora spiraeae*	CFCC 50049^T^	MG707859	MG708196	MG708199		
*Cytospora spiraeae*	CFCC 50050	MG707860	MG708197	MG708200		
*Cytospora spiraeicola*	CFCC 53138^T^	MN854448		MN850749	MN850756	MN861118
*Cytospora spiraeicola*	CFCC 53139	MN854449		MN850750	MN850757	MN861119
*Cytospora tamaricicola*	CFCC 50507	MH933651	MH933559	MH933616	MH933525	MH933587
*Cytospora tamaricicola*	CFCC 50508^T^	MH933652	MH933560	MH933617	MH933523	MH933588
*Cytospora tanaitica*	MFLUCC 14-1057^T^	KT459411	KT459413			
*Cytospora thailandica*	MFLUCC 17-0262^T^	MG975776	MH253459	MH253463		
*Cytospora thailandica*	MFLUCC 17-0263^T^	MG975777	MH253460	MH253464		
*Cytospora tibetensis*	CF 20197026	MK673076	MK673046	MK673016	MK672962	MK672992
*Cytospora tibetensis*	CF 20197029	MK673077	MK673047	MK673017	MK672963	MK672993
*Cytospora tibouchinae*	CPC 26333^T^	KX228284				
*Cytospora translucens*	CXY 1351	KM034874				KM034895
*Cytospora translucens*	CXY 1359	KM034871				KM034894
*Cytospora ulmi*	MFLUCC 15-0863^T^	KY417759				
*Cytospora verrucosa*	CFCC 53157^T^	MW418408		MW422911	MW422923	MW422935
*Cytospora verrucosa*	CFCC 53158	MW418410	MW422901	MW422913	MW422925	MW422937
*Cytospora vinacea*	CBS 141585^T^	KX256256			KX256277	KX256235
*Cytospora viridistroma*	CBS 202.36^T^	MN172408			MN271853	
*Cytospora viticola*	Cyt2	KX256238			KX256259	KX256217
*Cytospora viticola*	CBS 141586^T^	KX256239			KX256260	KX256218
*Cytospora xinjiangensis*	CFCC 53182	MK673064	MK673034	MK673004	MK672951	MK672980
*Cytospora xinjiangensis*	CFCC 53183^T^	MK673065	MK673035	MK673005	MK672952	MK672981
*Cytospora xinglongensis*	CFCC 52458	MK432622	MK442946	MK578082		
*Cytospora xinglongensis*	CFCC 52459	MK432623	MK442947	MK578083		
*Cytospora xylocarpi*	MFLUCC 17-0251^T^	MG975775	MH253458	MH253462		
*Cytospora zhaitangensis*	CFCC 56227^T^	OQ344750	OQ410623	OQ398733	OQ398760	OQ398789
*Cytospora zhaitangensis*	CFCC 57537	OQ344751	OQ410624	OQ398734	OQ398761	OQ398790
*Diaporthe vaccinii*	CBS 160.32	KC343228	JQ807297		KC343954	KC344196

Note. Ex-type strains are marked with T and isolates from the present study are in bold.

## Data Availability

All sequence data are available in NCBI GenBank (Table 1).
